# Potential benefits of student- and junior doctor-led textbooks

**DOI:** 10.1007/s40037-015-0185-9

**Published:** 2015-05-07

**Authors:** Zeshan U Qureshi, Katherine Lattey, Patrick Bryne, Mark Rodrigues, Michael Ross, Simon Maxwell

**Affiliations:** 1 Great Ormond Street and Institute of Child Health, Honorary Clinical Tutor, University of Edinburgh, Edinburgh, UK; 2 25 Joseph Hardcastle Close, SE14 5RN, New Cross, UK; 3 Brighton and Sussex Medical School, Brighton, UK; 4 Belford Hospital, Belford, UK; 5 University of Edinburgh, ST3 Radiology, Royal Infirmary of Edinburgh, Edinburgh, UK; 6 University of Edinburgh, Edinburgh, UK; 7 Clinical Pharmacology Unit, Clinical Research Centre, Western General Hospital, University of Edinburgh, Edinburgh, UK

**Keywords:** Medical education, Textbooks, Junior doctors, Medical students, Near peer teaching

## Abstract

**Introduction:**

Medical textbooks are an important teaching supplement. Few have junior doctors or medical students (‘juniors’) as primary contributors. However, the strengths of junior-led face-to-face teaching are now well-established, and we hypothesized that similar advantages would be transferrable to a textbook setting.

**Methods:**

Juniors were approached to contribute to an independently published medical textbook, with senior clinicians recruited in parallel to ensure factual accuracy. Juniors directed every aspect of textbook writing and the production process. The published book stressed that it was an open collaboration with readers, inviting them to get in touch to evaluate the text and suggest ideas for new titles.

**Results:**

Of 75 respondents, 93 % awarded the first textbook in the series 4 or 5 out of 5 for overall quality. Five other titles have been released, with seven more in development. Over 100 juniors are currently involved, with two students progressing from reviewers to editors after less than a year of mentorship.

**Conclusion:**

Juniors can be a motivated, dynamic, innovative group, capable of significant contributions to the medical textbook literature. This initiative has generated a sustainable infrastructure to facilitate junior-led publishing, and has the capacity for expansion to accommodate new initiatives and ideas.

## Introduction

The literature supports our own experience that juniors can be effective medical teachers [1, 2], and can use their recent experience of being learners to relate to current students and help them identify and address their learning needs. Under certain circumstances their teaching has compared very favourably to that delivered by senior staff [3, 4], and may have advantages such as how approachable they seem to students [5]. We hypothesized that similar advantages may equally apply to the writing of textbooks. Such resources could complement core medical school teaching, and senior doctor-led textbooks. Contributing to the writing of textbooks may also offer an opportunity for juniors to be mentored, develop their writing skills, and consolidate medical knowledge.

Few publishing groups directly recruit junior doctors or medical students (juniors) to write textbooks, and we are not aware of any others which use them as their primary base of authors and editors. We developed a new approach to developing and publishing medical textbooks which explicitly seeks to do exactly that. The first textbook in the series, ‘The Unofficial Guide to Passing OSCEs’, involved 37 juniors as authors and editors, and 38 experts who checked the content for accuracy [6]. The book has been distributed to approximately 8000 people in 40 countries. Five further titles have now been released, with another seven in development, involving a team of over 100 juniors worldwide. This article describes the approach taken to developing these textbooks, including the recruitment of juniors, collaboration with experts and students, and plans for evaluation and future development.

## The process of recruiting juniors

All interested juniors are given an open invitation to offer feedback on current and possible future textbooks. This allows a measure of their level of commitment and their ability to contribute. Those who show insight, enthusiasm and organization progress to a junior reviewer role for material in a new textbook, where they start to gain insight into the textbook production process. Junior reviewers work closely with the editor to ensure material is relevant to the curriculum, addresses their key learning needs, and is expressed clearly. Subsequently, juniors may be promoted to authors. Authors are provided with templates to follow, and are initially responsible for drafting short sections of a textbook, with support from expert reviewers and editors. Expert reviewers are carefully selected to ensure content is in line with current evidence and practice.

Successful authors may then progress to editors, who develop a textbook idea and structure, and coordinate the development of the textbook, from managing authors and reviewers, to managing the graphic design and print review process.

This process allows juniors with no experience in textbook writing to rapidly develop the necessary skills to take on more demanding roles.

## Collaboration with senior clinicians

The content of the textbooks is at the level expected of medical undergraduates, and as such juniors who have passed, or are revising for, relevant exams should have a sufficient level of understanding to inform their teaching on those topics. However, they will not have the depth of understanding and experience of an expert, the value of which cannot be underestimated. The challenge is how best to involve senior staff without losing the benefits that come from having material created from the junior perspective. We have addressed this by ensuring that senior clinicians approve content for factual accuracy at multiple stages of the textbook production process, without directly writing any of the content for the textbooks.

## Collaboration with medical students

There is usually a clear distinction between the authors of a medical textbook and the readers. Authors are typically senior clinicians who impart their wisdom, and readers are the ‘learners’ who benefit from that wisdom. This project aimed to blur this distinction, by giving readers agency, and actively encouraging them to engage with, and contribute to, the development of their textbooks. Feedback is traditionally gathered from students after publishing, but unusually we also chose to integrate dialogue with students into the textbook production process.

Provisional ideas for textbooks are developed with input from the student community at an early stage. One route is via social media, which permits close interaction with a large and diverse group of students—something not previously possible. We have a Facebook group, ‘The Unofficial Guide to Medicine’, with over 23,500 members [7]. They were asked, for example, to review draft contents for a radiology textbook, and suggested an additional five chapters which were subsequently written before the textbook went to press. Textbook content is therefore reviewed at multiple stages by both experts and student groups. Juniors have been involved in every aspect of the development process of textbooks, from suggesting new titles, to reviewing content, to developing graphic design and appraising the final printed product pre-release. This makes the production of new textbooks an iterative process, with the content and design of textbooks being adjusted at multiple stages, until the desired outcome is reached.

## Key differences in junior-led textbooks

With the textbooks being junior-led at every stage, we think the content of the textbooks is likely to better reflect student needs, compared with student needs as they are indirectly perceived by a publishing company. Another key difference is the simplicity of the language, and the friendly tone of the textbooks, potentially making the textbooks more accessible, and easier to read. Junior input into the textbook production process has also had a significant impact on the structure, page layout, and graphic design. Textbooks so far have typically included high-quality illustrations, with additional space to take notes and signposting for easier navigation. We have been willing to continually improve and add content to any textbook throughout the production cycle, as long as it seemed to be responding to student needs as expressed by the reviewers or via social media.

Involving many juniors at each stage, with the power to cause a significant change in content, can of course have potential negative effects, such as delayed publication, increased workload for writers, and increased costs—particularly for alterations after graphic design or printing. Such feedback is valuable, however, even at this late stage, as certain issues may only become apparent after page layout has been drafted (e.g. images being too small).

## Direct evaluation of the effect on student learners and contributors

Several studies have looked at the benefits of junior-led teaching in undergraduate medical education [8]. A 2011 systematic review of 19 studies assessing medical students-as-teachers concluded that peer teaching in undergraduate medical teaching was comparable with conventional teaching in selected contexts [9]. One study found that prescribing skills when formally examined were better in those who attended junior doctor-led prescribing teaching than those who did not attend [10]. Benefits for those who deliver the teaching are also clear, for example improving teaching skills and clinical knowledge [5].

There has been no published research to date on the benefits of junior-led textbooks, and thus the benefits suggested herein are based on extrapolation from the broad benefits of junior-led teaching.

Readers of the first textbook we produced (purchased via amazon.co.uk) were invited to rate it on a five-point scale, and give qualitative feedback [11]. Of the 75 respondents, 93 % awarded it four or five for overall quality. The qualitative feedback that we have received from contributors and readers is summarized in Table 1, and is largely positive.

**Table 1:** Potential benefits and harms from junior-led

	Contributors	Readers
**Potential benefits**	‘Helps strengthen publication CV’	‘Formatted in a similar way to how most people make notes themselves—concise, bullet-pointed and logically ordered, so a great time-saver!’
	‘Helps to learn new topics and practice teaching by way of writing’	
	‘Develops ability to communicate complex ideas, and to work with colleagues from different backgrounds’	‘The multi-author collaborative approach works exceptionally well and the democratisation of the reviewing process ensures that this will meet the needs of medical students and junior doctors, both in their exams and in their day to day work’
	‘Quite rewarding, seeing the process through the various stages to getting the final product in your hand and knowing that you've contributed to it, that people will actually buy it because it is a high quality product’	‘This has all the information of a textbook without being as difficult to read’
	‘Being mentored by other juniors that have gone through the same process’	‘This guide sets out the basics of how to do examinations, histories, etc. without discombobulating the reader. It does this by using direct language and clear formatting while not scrimping on important minutiae’
	‘An opportunity to gain an extraordinary sense of achievement, and reach students not just on your ward, but also in other countries’	
	‘A very useful way of getting practice writing in a scientific style and engaging with clinical topics; writing the chapters forces you to really understand a subject’	
**Potential harms**	‘As a student I have found that sometimes my clinical knowledge is lacking compared with a junior doctor. Meaning that sometimes the drafts I have submitted have been rewritten quite heavily’	‘Doesn't go into enormous amounts of detail so it complements other textbooks, rather than being a stand-alone guide to clinical examination. Nevertheless it is a decent textbook that I will consult during the remainder of my undergraduate education’
	‘Time consuming’	
	‘Lots of re editing’	
		‘Doesn't go into a lot of depth’

For contributors, the time commitment and the challenge of developing material to a publishable level are clear potential difficulties. However, benefits may be accrued from receiving mentorship, improving teaching skills, improving clinical knowledge, and feeling rewarded for what is perceived as an activity with significant educational value. Two reviewers have progressed to book editors in less than a year of mentorship, highlighting value in terms of professional development.

Readers emphasize the ease of use, the relevance of content, and the value of a multi-author collaborative approach as advantages. One potential criticism has been a focus on the textbooks summarizing topics, rather than detailed explanations. This re-emphasizes the importance of junior-led teaching complementing, but not replacing, that provided by senior clinicians.

Further and more detailed evaluation is needed for more substantial conclusions to be drawn. We are currently formally assessing the effects of junior-led textbooks on the professional development of writers, and the value of junior-led textbooks, compared with senior-led textbooks, from a student perspective.

## Conclusion

Medical students and junior doctors can be a motivated and hardworking group, capable of significant contributions to the medical textbook literature. This project has set up a sustainable infrastructure to facilitate junior-led publishing, with user feedback at multiple stages in the process and quality control through expert review, and has the capacity to expand and accommodate new initiatives and ideas. Further research is ongoing, more formally evaluating the benefits of this process for contributors and readers alike.
